# The Diacylglycerol Acyltransferase 3 of *Chlamydomonas reinhardtii* Is a Disordered Protein Capable of Binding to Lipids Derived from Chloroplasts

**DOI:** 10.3390/biom15020245

**Published:** 2025-02-08

**Authors:** Natalia Pavia, Alberto Potenza, Felipe Hornos, José A. Poveda, Gabriela Gonorazky, José L. Neira, Ana M. Giudici, María Verónica Beligni

**Affiliations:** 1 Instituto de Investigaciones Biológicas (IIB-CONICET-UNMdP), Facultad de Ciencias Exactas y Naturales, Universidad Nacional de Mar del Plata, Mar del Plata 7600, Argentina; nataliapavia28@gmail.com (N.P.); gonorazk@mdp.edu.ar (G.G.); 2 Instituto de Investigación, Desarrollo e Innovación en Biotecnología Sanitaria de Elche (IDiBE), Universidad Miguel Hernández, 03202 Elche, Spain; apotenza@umh.es (A.P.); fhornos@umh.es (F.H.); ja.poveda@umh.es (J.A.P.); jlneira@umh.es (J.L.N.); 3 Institute of Biocomputation and Physics of Complex Systems (BIFI), Universidad de Zaragoza, 50018 Zaragoza, Spain

**Keywords:** protein–lipid interactions, leakage, fluorescence, disordered protein, thylakoids

## Abstract

Understanding triacylglycerol (TAG) metabolism is crucial for developing algae as a source of biodiesel. TAGs are the main reservoir of energy in most eukaryotes. The final, rate-limiting step in the formation of TAGs is catalyzed by 1,2-diacylglycerol acyltransferases (DGATs). In the green alga *Chlamydomonas reinhardtii*, DGAT3 is phylogenetically related to plant DGAT3 but unrelated to other DGATs from eukaryotes, such as DGAT1 and DGAT2. In this study, we described the conformational preferences and the lipid-binding features of the DGAT3 from *C. reinhardtii*. To characterize its conformational stability and structural features, we used several biophysical probes, namely, fluorescence, circular dichroism (CD), and differential scanning calorimetry (DSC). Our results showed that the protein was mainly disordered, containing a small population of folded conformations in a narrow pH range (pH 8 to 10). The conformational stability of the folded structure of DGAT3 was very low, as shown by urea or guanidinium denaturations. Thermal denaturation, followed by fluorescence or CD, as well as calorimetric denaturation, followed by DSC, did not yield any transition in the pH range where DGAT3 acquired a “native-like” conformation. Furthermore, we used two approaches to demonstrate the interaction of DGAT3 with lipid membranes at the pH at which it had acquired a “native-like” conformation. The first involved the measurement of anisotropy and fluorescence quenching of the protein. The second approach focused on examining possible modifications of the biophysical properties of lipids due to their interaction with DGAT3, through anisotropy measurements and leakage assays. Both methods produced consistent results, suggesting that DGAT3 preferentially interacted with negatively charged membranes. These results will allow the design of a more efficient and stable DGAT3, as well as an in-depth understanding of how the metabolism of TAGs is accomplished in *C. reinhardtii*.

## 1. Introduction

The development of sustainable sources for biodiesel production has sparked the study of eukaryotic microalgae as feedstock for triacylglycerols (TAGs). One of the outcomes of these studies is that many species increase their TAG content under stress conditions and nutrient deprivation. However, TAG accumulation does concomitantly result in a reduction in cell proliferation, and therefore, in a decrease in the global amount of oil produced. Thus, genetic engineering and an in-depth understanding of the metabolism of lipids and their interactions with other molecules are necessary to tailor specific algae to synthesize oil in a sustainable, economically viable fashion. Our knowledge about TAG metabolism in algae is scarce and mostly inferred from plants, in those occasions in which there is evidence of similarities between microalgal and plant TAG metabolism [1,2].

TAGs are the main molecules involved in energy storage in most eukaryotes. There are several well-known pathways for TAG synthesis. In the best characterized one (the Kennedy pathway [3]), there is a sequential acylation of fatty acids, mostly in the form of acyl-CoA, on a glycerol-3-phosphate backbone. This pathway—in plants, yeast, and animals, where it has been fully described—occurs in the endoplasmic reticulum [4,5]. The final rate-limiting step of this pathway, acylation of acyl-CoA onto a molecule of sn-1,2-diacylglycerol (DAG) [6], is catalyzed by several types of 1,2-diacylglycerol acyltransferases (DGATs). In mammals and plants, most DGATs are integral microsomal membrane proteins [5,7], but little is known about the possible presence, function, and structure of these kinds of proteins in algae.

Recent data-mining and phylogenetic analyses identified a soluble DGAT, exclusive to green algae and moderately related to a plant homolog, the so-called DGAT3 [8,9]. The DGAT3 clade has a most recent ancestor in common with a group of non-characterized polypeptides from cyanobacteria, probably from a prokaryotic origin [9,10]; in contrast, canonical DGATs, DGAT1 and DGAT2, are only related to eukaryotic proteins. DGAT3 has been characterized in only a few species of plants (see [11] and references therein). In all examples described so far, it has a C-terminal iron–sulfur (2Fe-2S) cluster-binding domain with a thioredoxin-like fold, which is also observed in DGAT3 from *Arabidopsis thaliana* [12]. In addition, most algal sequences within the DGAT3 group have been predicted to localize to the chloroplast [9], and this has been empirically confirmed for the *C. reinhardtii* protein [11]. The DGAT3 from *C. reinhardtii*, when expressed in *Escherichia coli*, produces an increase in the amount of TAG in the presence of oleate [9]. Initial sequence analyses of *C. reinhardtii* DGAT3 show the lack of transmembrane segments and content in hydrophobic residues lower than in the canonical members of the family [9]; those features seem to be also found in DGAT3 from plants [13,14,15]. Taken together, these findings suggest that DGAT3 from *C. reinhardtii* could be involved in a soluble TAG synthesis pathway in the chloroplast [9,11]. Although there are some recent descriptions of the function of DGAT3 in several plants [12,16], there are no studies describing its conformational features and its ability to bind to lipids in vitro.

In this work, we purified the DGAT3 of *C. reinhardtii* to: (i) allow for a structural and conformational characterization of the isolated protein in solution, and (ii) describe its interaction with lipids. Our findings, obtained by using fluorescence, circular dichroism (CD), and differential scanning calorimetry (DSC), indicated that DGAT3 from *C. reinhardtii* was mainly disordered, although it contained a population of ordered structure (probably, a β-sheet-like conformation), as concluded from CD spectra deconvolution. This secondary structure was not stable against temperature, as shown by spectroscopic thermal denaturations, followed by fluorescence and CD, and by DSC. However, this secondary structure seemed to unfold co-operatively in the presence of urea and guanidine hydrochloride (GdmCl). Thus, we can conclude that the secondary structure of *C. reinhardtii* DGAT3 was not rigid enough. In addition, we also employed biophysical experimental approaches to investigate the interaction of DGAT3 with membranes. Analysis of anisotropy and fluorescence quenching of the protein, both in the presence and absence of a range of liposomes designed to mimic different cellular membranes, revealed a preferential interaction with those containing the anionic phospholipid PG, and also with membranes resembling those of chloroplast thylakoids. Similar results were obtained when carrying out leakage assays with DGAT3 and the liposomes. In contrast, DGAT3 had minimal effects on vesicles containing mostly zwitterionic phospholipids. The possible relevance of these results in the context of *C. reinhardtii* DGAT3 sequence, localization, and regulation is discussed.

## 2. Materials and Methods

### 2.1. Materials

Imidazole, Trizma acid and its sodium salt, boric acid, molecular biology-grade urea, acetic acid and its sodium salt, phosphoric acid and its three sodium salts, Hepes acid and its sodium salt, Roche protease tablets, NaCl, Ni^2+^-resin, EDTA, ultra-pure Triton X-100, β-mercaptoethanol, standard molecular markers, and Amicon centrifugal devices with a molecular weight cut-off of 30 kDa were obtained from Merck (Madrid, Spain). Kanamycin and isopropyl-β-D-1-thiogalactopyranoside (IPTG) were obtained from Apollo Scientific (Stockport, UK). Triton X-100, TCEP (tris(2-carboxyethyl)phosphine), and the SDS protein marker (PAGEmark Tricolor) were obtained from VWR (Barcelona, Spain). Ultra-pure urea and GdmCl were obtained from MP Biomedicals LLC (Solon, OH, USA).

Dimyristoylphosphatidylcholine (DMPC); dimyristoylphosphatidylglycerol (DMPG); 1′,3′-bis [1,2-dioleoyl-sn-glycero-3-phospho]-glycerol, sodium salt (18:1 cardiolipin, CL); L-α-phosphatidylglycerol (soy PG); 1-palmitoyl-2-oleoyl-sn-glycero-3-phospho-(1′-rac-glycerol (POPG); L-α-phosphatidylcholine from egg chicken (PC); chicken egg sphingomyelin (SM); bovine liver phosphatidylethanolamine (PE); 1-palmitoyl-2-oleoyl-glycero-3-phosphocholine (POPC); ovine wool cholesterol (CHO); porcine brain L-α-phosphatidylserine (PS); plant monogalactosyldiacylglycerol (MGDG); plant digalactosyl-diacylglycerol (DGDG); and sulphoquinovosyl-diacylglycerol (SQDG) were obtained from Avanti Polar Lipids (Alabaster, AL). The 1,6-diphenyl-1,3,5-hexatriene (DPH), and 1-(4-trimethylammoniumphenyl)-6-phenyl-1,3,5-hexatriene (TMA-DPH) were obtained from Molecular Probes (Thermo Fisher Scientific, Madrid, Spain).

### 2.2. Protein Expression and Purification

The codon-optimized, N-terminal His-tagged *dgat3* gene inserted into the vector pET30a(+) (with kanamycin resistance) was synthesized, produced, and purified by GenScript (Piscataway, NJ, USA). The coding sequence was introduced between the positions NdeI and HindIII of the vector. The sequence of the expressed protein is shown in Figure S1.

Expression of DGAT3 was carried out in *E. coli* BL21 (DE3) strain (Merck, Madrid, Spain), after chemical transformation with the vector indicated above. A single colony of transformed cells was used to inoculate a starter culture (100 mL de Luria-Bertani (LB) media: 10 g/L of tryptone, 5 g/L of yeast extract, and 10 g/L of NaCl) containing a final concentration of 30 μg/mL of kanamycin; the starter culture was grown at 37 °C overnight with shaking (125 rpm). The next day, this overgrown culture was used to inoculate 3 × 1 L of LB media containing kanamycin (30 μg/mL) in a 2-L flask. The cell culture was grown at 37 °C with shaking (220 rpm) until the absorbance at 600 nm of LB medium reached a value in the range of 0.8–1.0, with a final amount of 0.4 mM of IPTG. After induction, cells were grown overnight at 25 °C and harvested by centrifugation at 5 °C for 15 min at 7000 rpm in a JA-10 rotor (using a Beckman Coulter Avanti J26-XP Centrifuge, Barcelona, Spain). The cell pellet of the 3 L of LB media was resuspended in 50 mL of lysis buffer, containing 50 mM Tris, 1% Triton X-100, 500 mM NaCl, 1 mM β-mercaptoethanol, and 10 mM imidazole, pH 8.0 (buffer A), with an additional Roche protease inhibitor cocktail EDTA-free tablet. The cell suspension was lysed by sonication on ice with 10 bursts of 45 s at maximum power, interleaved with 15 s keeping the samples on ice (the sonicator was a Branson model 102C). Insoluble cell debris was removed from the lysate by centrifugation for 30 min at 17,000 rpm in a JA-20 rotor (using a Beckman Coulter Avanti J26-XP Centrifuge, Beckman Coulter, Barcelona, Spain) at 5 °C. In our first attempts to purify the protein from the soluble fraction at the lysis step, we observed that the percentage of DGAT3 in such a fraction was very small, and it was highly impure, with proteins of similar molecular weight. Furthermore, we could not re-purify the protein from the Ni-resin because it bound to all size exclusion columns available in our laboratories. We concluded that DGAT3 was mainly expressed as inclusion bodies.

To purify DGAT3 from the inclusion bodies, the pellet from the lysis homogenate was treated with buffer A plus 8 M urea, and the resulting suspension was sonicated on ice with 15 bursts of 45 s at maximum power interleaved with 15 s on ice. The insoluble cell debris was removed from the cell lysate by centrifugation for 30 min at 17,000 rpm in a JA-20 rotor (using a Beckman Coulter Avanti J26-XP Centrifuge, Barcelona, Spain) at 5 °C. The supernatant was loaded onto the HiTrap resin (5 mL) within a BioRad sleeve column, equilibrated with lysis buffer plus 8 M urea, and the flow-through was separated from the resin by gravity at room temperature. The resin was then washed with 100 mL of washing buffer, containing 50 mM Tris, 500 mM NaCl, 1 mM β-mercaptoethanol, and 30 mM imidazole, pH 8.0 (this procedure is called on-column refolding [17,18,19]) at room temperature. The DGAT3 protein was eluted with 10 mL of buffer containing 50 mM Tris, 500 mM NaCl, 1 mM β-mercaptoethanol, and 500 mM imidazole, pH 8.0 at room temperature. The protein was exchanged to buffer 50 mM Tris, 150 mM NaCl, and 5 mM TCEP, pH 8.0 by using Amicon Centrifugal devices of 30 K cut-off in an Allegra X-15 R BenchTop Centrifuge (Beckman Coulter, Barcelona, Spain), with an SX-4750 rotor at 3500 rpm for 5 changes of 15 mL of buffer each at 5 °C. The purity of the protein was checked by 12% SDS-PAGE gels, after eluting it from the resin, and after the buffer exchange step, and it was in all cases higher than 85%. We tried to polish the eluted protein from the Ni-resin by using a gel filtration Superdex 200 16/60 size-exclusion column (GE Healthcare, Madrid, Spain) equilibrated in 50 mM Tris buffer and 5 mM TCEP pH 8.0, by using a range of 150 to 300 mM of NaCl, working on an AKTA FPLC (GE Healthcare) by following the absorbance at 280 nm. At all the explored NaCl concentrations, DGAT3 was bound to the column.

Protein concentration was estimated by measuring the absorbance at 280 m with the extinction coefficients as determined from the sequence (two tryptophans and one tyrosine) [20]. The protein yield was 1.5–2.0 mg/L of culture. The protein was flash-frozen in liquid nitrogen and stored at −20 °C in the above buffer for further use.

### 2.3. Sample Preparation for Studies in the Presence of Lipids

Aliquots with the corresponding amount of lipid in CHCl_3_/CH_3_OH (1:1, *v*/*v*) were placed in a test tube. Both solvents were removed by evaporation under a stream of O_2_-free nitrogen, and their traces were eliminated under a vacuum in the dark for more than 3 h. The solution containing the protein was then added to the dried lipid to obtain the required specific lipid/protein molar ratio. The resulting suspension was vortexed at ~5 °C above the transition temperature of the phospholipid to obtain multilamellar vesicles (MLV). The mixture was frozen/thawed twice to ensure complete sample homogenization and maximization of contacts between the protein and the phospholipid. The resulting sample was incubated for 10 min at ~5 °C above the transition temperature of the phospholipid, with occasional vortexing.

For fluorescence anisotropy experiments using either DPH or TMA-DPH, both MLVs containing DGAT3 and controls without protein were used. The buffer composition was 50 mM Tris, 100 mM NaCl, and 5 mM TCEP, pH 8. The proper amount of a stock solution of DPH or TMA-DPH at a 1.28 mM concentration in dimethylformamide was added to the MLV suspension. The resulting solution was incubated at 55 °C for 60 min for DPH, and for 20 min for TMA-DPH-containing liposomes, respectively. The lipid/protein molar ratio was 100:1, and the fluorescence probes/lipid molar ratio was 1:500.

For quenching assays in the presence of membrane models or vesicle leakage experiments, large unilamellar vesicles (LUVs) were prepared by the extrusion method using a 100-nm pore-size membrane. The proper amounts of lipid in CHCl_3_ were mixed and dried by N_2_ gas. Next, the CHCl_3_ was completely removed by placing the sample in a vacuum desiccator for more than 3 h. Then, 1 mL of buffer (10 mM Tris, 20 mM NaCl, 40 mM Carboxifluorescein (CF), 0.1 mM EDTA, pH 7.4) was added to the dry phospholipid mixture, and MLVs were obtained by vortexing at room temperature. In the next step, we performed five freezing/thawing cycles of the MLV solution; it was frozen in liquid N_2_ for 2 min, and then thawed at room temperature for 20 min. The resulting solution was extruded through a 100-nm pore-size membrane using the LiposoFast apparatus (Avanti polar lipid, Sigma-Aldrich, Madrid, Spain) until it became transparent. Non-encapsulated CF was separated from the vesicle suspension by using a Sephadex G-75 filtration column (Pharmacia, Uppsala, Sweden) running with a buffer containing 10 mM Tris, 100 mM NaCl, 0.1 mM EDTA, pH 7.4. For quenching experiments, LUVs were obtained with the same procedure, but a 50 mM Tris, 100 mM NaCl, pH 8 buffer was employed to resuspend the dry phospholipid mixture.

The lipid compositions of membrane models used in our studies were as follows: *Staphylococcus aureus* membrane is composed of 42% CL and 58% PG [21]; eukaryotic plasma membrane is composed of 25% PC, 18% SM, 13% PE, 8% PS, 36% CHO [22]; *Escherichia coli* membrane is composed of 80% PE, 15% PG, and 5% CL [23]; thylakoid membrane is composed of 40% MGDG, 30% DGDG, 16% SQDG and 14% POPG [24].

### 2.4. Fluorescence

Spectra were collected at 25 °C using a Cary Varian spectrofluorimeter (Agilent, Santa Clara, CA, USA), with a Peltier temperature controller. Sample concentrations were 3 μM, and that of the corresponding buffer was, in all cases, 50 mM. The experiments were conducted as previously described [25,26], using a 1 cm-pathlength quartz cell (Hellma, Müllheim, Germany).

#### 2.4.1. Intrinsic Fluorescence

Protein samples were excited at 280 and 295 nm across the pH range from 2.0 to 12.0, to account for possible differences in the behavior of the two tryptophans and the single tyrosine in the DGAT3 sequence (Figure S1). The rest of the experimental setup has been described elsewhere [25,26]. Suitable blank corrections were made in all spectra.

Chemical denaturations at pH 8.0 (50 mM Tris, 150 mM NaCl, and 5 mM TCEP were performed using fluorescence or CD, as described elsewhere [25,26].

The pH of each sample was measured after completion of the experiments with an ultra-thin Aldrich electrode in a Radiometer pH meter (Radiometer Medical ApS, Brønshøj, Denmark). The salts and acids used have been described elsewhere [25,26]. Chemical and pH denaturations were repeated three times with new protein samples.

The wavelength averaged emission intensity (also called the spectrum mass center), <λ>, was calculated as described [27]. The <λ> is defined as: $<\lambda >\;=\;\sum_{1}^{n}\frac{1}{\lambda_{i}}I_{i}/\sum_{1}^{n}I_{i}$, where *I*_i_ is the intensity at wavelength λ_i_. We shall report <λ> in units of μm^−1^.

#### 2.4.2. Thermal Denaturation Experiments

Thermal scans were collected at 330 or 350 nm after excitation at 280 or 295 nm from 25 to 85 °C with heating rates of 60 °C/h. The rest of the experimental setup was the same as described above, with additional details described elsewhere [25,26]. The thermal denaturation for DGAT3 was not reversible at any of the explored pH values.

#### 2.4.3. Fluorescence Quenching

Quenching of the fluorescence by KI was examined under denaturant conditions and at different pH values. Protein concentrations were 4 μM in all cases. Excitation was 280 or 295 nm. The rest of the experimental details have been described elsewhere [25,26]. The data were fitted to [28]:

(1) F0F=1+KsvX

where *K*_sv_ is the Stern–Volmer constant for collisional quenching; *F*_0_ is fluorescence when no KI is present; and *F* is the fluorescence at any KI concentration (between 0 and 0.6 M). We explored the quenching at pH 3.3 (acetate buffer), pH 8.1 (Hepes buffer), and 12.2 (boric buffer). Experiments were also carried out in the presence of 4 M GdmCl at pH 8.1 (50 mM Hepes buffer).

Fluorescence quenching by iodide was also examined in the presence of membrane models. Protein concentrations were 5 μM and LUV concentrations were 0.1 mM.

#### 2.4.4. ANS Binding

ANS was used to monitor the pH denaturation of DGAT3 (at 4 μM concentration). The experimental details have been described elsewhere [25,26]. The final ANS concentration was 100 μM in all the DGAT3 samples. Blank solutions were subtracted from the corresponding spectra.

Thermal denaturation was also monitored with an excitation wavelength of 380 nm, and emission was collected at 490 and 510 nm. The rest of the parameters were the same as those used in the intrinsic fluorescence experiments.

#### 2.4.5. Steady-State Anisotropy Fluorescence Measurements

The steady-state fluorescence anisotropy of DGAT3, <*r*>, was measured with a PicoQuant F300 (PicoQuant, Berlin, Germany) spectrofluorometer and calculated as [28]:

(2) $$<r>\;=\;\left(I_{VV}-GI_{VH}\right)/\left(I_{VV}+2GI_{VH}\right)$$

where *I*_VV_ and *I*_VH_ are the fluorescence intensities (blank subtracted) of the vertically and horizontally polarized emission when the sample is excited with vertically polarized light, respectively. The *G*-value corresponds to the instrument correction factor (*G* = *I*_VH_/I_HH_). The samples were measured at 340 nm after excitation at 295 nm. A final protein concentration of 5 μM in buffer 50 mM Tris, 100 mM NaCl, 5 mM TCEP, pH 8 was used. Ten measurements were taken for each sample to calculate the average steady-state anisotropy values (±standard deviation).

For some lipids, the variation in <*r*> was fitted to [29]:

(3) $$<r>\;=\;\left(r_{W}D+r_{L}YK_{p}\right)/\left(YK_{p}+D\right)\;\;$$

from which the partition coefficient of the protein in the lipid milieu was calculated as: $K_{p}=\left(n_{L}/V_{L}\right)/\left(n_{W}/V_{W}\right)$, where *n*_i_ stands for the moles of peptide in phase i and *V*_i_ represents the volume of phase i, with phase i being either aqueous (i = W) or lipidic (i = L); *r*_W_ and *r*_L_ are the steady-state anisotropies in the aqueous and lipid phases, respectively; *Y* = Φ_L_/Φ_W_ is the ratio of the fluorescence quantum yield of the polypeptide in the lipid and aqueous phases; and *D* = (1/γ[L]) − 1 is a function of [L], the lipid concentration, and γ, the molar volume of the lipid (0.7 M^−1^).

### 2.5. Membrane Leakage Measurements

Membrane rupture (leakage) of intraliposomal CF was tested by exposing the probe-loaded liposomes (final lipid concentration, 0.125 mM) with the appropriate amounts of DGAT3 on microtiter plates stabilized at 25 °C using a microplate reader (POLAstar, BMG Labtech, Ortenberg, Germany). Each well contained a final volume of 170 μL. The medium in the microtiter plates was continuously stirred to allow rapid mixing of DGAT3 and the vesicles. Leakage was measured at an approximate DGAT3-to-lipid molar ratio of 1:100 and 1:50. Changes in fluorescence intensity were recorded with excitation and emission wavelengths set at 492 and 517 nm, respectively. Fluorescence measurements were taken initially with probe-loaded liposomes, followed by the addition of DGAT3 solution, and finally adding Triton X-100 to obtain 100% leakage. Leakage was quantified on a percentage basis (% release) according to the equation:

(4) $$\%\;release=\left(\left(F_{t}-F_{0}\right)/\left(F_{100}-F_{0}\right)\right)\times 100$$

where *F*_t_ is the equilibrium value of fluorescence 15 min after DGAT3 addition, *F*_0_ is the initial fluorescence of the vesicle suspension, and *F*_100_ is the fluorescence value after the addition of Triton X-100 (to a final concentration of 1% (*w*/*w*) [30]).

### 2.6. Circular Dichroism (CD)

Circular dichroism spectra were collected on a Jasco J810 (Tokyo, Japan) spectropolarimeter fitted with a thermostated cell holder and interfaced with a Peltier unit. Molar ellipticity was calculated as described previously [25].

#### 2.6.1. Far-UV Spectra

Isothermal wavelength spectra of DGAT3 at different pHs and GdmCl or urea concentrations were prepared, acquired, and corrected as described elsewhere [25,26]. The protein concentration was 10 μM in 50 mM buffer.

#### 2.6.2. Thermal Denaturation Experiments

The experiments were performed as described elsewhere [25,26], with a total protein concentration of 10 μM. Solution conditions were the same as those reported in the steady-state experiments. Thermal denaturations were not reversible at any pH for DGAT3, as shown by: (i) spectral comparison before and after the heating, and (ii) changes in the voltage of the instrument as the temperature was raised [31].

### 2.7. Analysis of the pH, Thermal and Chemical Denaturation Curves

The pH denaturations were analyzed, when possible, if DGAT3, protonated and deprotonated, contributed to the spectral properties:

(5) $$X=\frac{\left(X_{a}+X_{b}10^{\left(n(pH-pK_{a}\right)}\right)}{\left(1+10^{\left(n(pH-pK_{a}\right)}\right)}$$

where *X*_a_ and *X*_b_ represent the observed spectral property (fluorescence intensity at any wavelength, ellipticity, or <λ>) at acid or basic pH values, respectively; p*K*_a_ is the apparent midpoint of the titrating group; and *n* is the Hill coefficient (which was close to 1 in all the curves reported in this work). The apparent p*K*_a_-values reported (from intrinsic or ANS fluorescence, and CD) were obtained from three different measurements in each technique, and prepared with new samples. For the different p*K*_a_-values obtained in the results of each technique, different regions were selected by visual inspection, and the data in that region were fitted to Equation (5).

The thermal and chemical denaturation data for DGAT3, when possible, were fitted to the two-state equation:

(6) X=XN+XDe−∆GRT1+e−∆GRT

where *R* is the gas constant, *T* is the temperature in K, and Δ*G* is the denaturation free energy. The *X*_N_ and *X*_D_ correspond to the physical property of the native and denatured DGAT3, respectively.

Chemical denaturation curves for DGAT3 were analyzed, when possible, using the linear extrapolation model (LEM), where the free energy, ΔG, is: ΔG = *m*([D]_1/2_ − [D]) [32], where [D] is the denaturant concentration, [D]_1/2_ is that at the midpoint of the transition, and *m* is the slope of the curve.

### 2.8. Differential Scanning Calorimetry

DSC experiments were performed on a Nano DSC differential scanning microcalorimeter (TA Instruments, New Castle, DE, USA) with capillary tantalum cells with a total volume of 0.3 mL.

Before the measurements, DGAT3 was centrifuged at 12,000× *g* for 10 min to remove any possible aggregates. The buffer used was 50 mM Tris, 150 mM NaCl, and 5 mM TCEP, pH 8.0. The solution of DGAT3 was degassed with gentle stirring in an evacuated chamber for 15 min at room temperature. The solution was loaded into the calorimetric cell after degassing. The reference cell was filled with the buffer. In both cells, a pressure of 3 atm of dry nitrogen was always kept throughout the scans to prevent any degassing during heating. The DGAT3 concentration was 0.5 mg/mL (~13.3 μM) and the scan rate used was 1.5 °C/min. A background scan collected using buffer in both cells was subtracted from the scan acquired with the protein. The excess molar heat capacity of DGAT3 was plotted vs. temperature by using the software (NanoAnalyze Data Analysis Version 3.8.0) package supplied by the instrument.

## 3. Results

### 3.1. DGAT3 Acquired a “Native-like” Conformation in a Narrow pH Range

To measure the conformational stability and the lipid-binding features of DGAT3, we must firstly determine in which pH range the protein acquired a “native-like” conformation. To that end, we used several biophysical probes. The whole set of techniques gives complementary information on different conformational features of DGAT3. We used intrinsic fluorescence to monitor changes in the tertiary structure of DGAT3 around its single tyrosine and the two tryptophans. We used ANS fluorescence to monitor the burial of solvent-exposed hydrophobic patches in DGAT3 (and to detect the presence of possible partially folded species [33]). Finally, we carried out far-UV CD experiments to monitor changes in the secondary structure of DGAT3.

#### 3.1.1. Fluorescence

**Figure 1:**
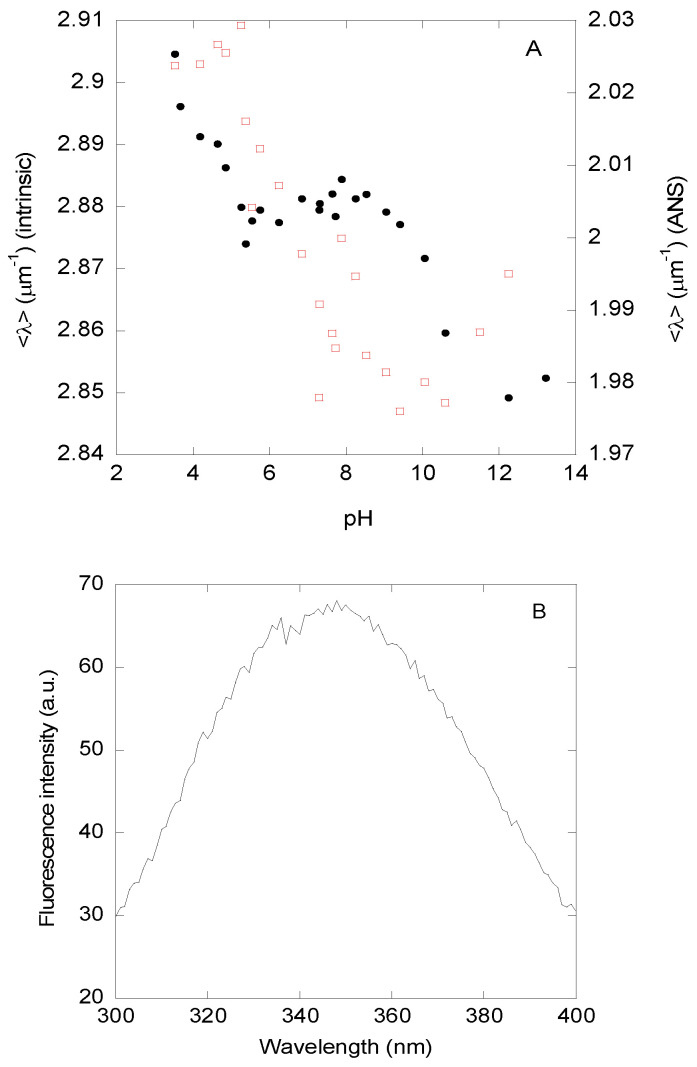
pH-induced structural changes in DGAT3 followed by fluorescence. (**A**) Changes in <λ> of the intrinsic fluorescence after excitation at 280 nm as the pH of the solution was varied, monitored by intrinsic (filled black circles, left axis) or ANS (blank red squares, right axis) fluorescence. (**B**) Fluorescence spectrum of DGAT3 at pH 8.1 after excitation at 280 nm. Experiments were carried out at 25 °C.

(1) *Steady-state fluorescence and thermal denaturations*—The <λ> (Figure 1A, filled circles) and the fluorescence intensity at 330 nm (Figure S2) of DGAT3 showed three transitions (after excitation at 280 nm, with similar results observed after excitation at 295 nm). The first occurred at acidic pHs, but we could not determine the p*K*_a_-value because of the absence of an acidic baseline. The second one occurred between pH values of ~5.0 and ~7.5; fitting the data in that pH range to Equation (5) led to a p*K*_a_-value of 6.2 ± 0.3. Finally, the third one occurred at basic pHs, but we could not determine the midpoint of the transition because of the absence of a basic baseline; this titration was probably due to the release of the phenolic proton of the single Tyr294. These results suggest that the protein acquires a “native-like” conformation between pH 8 and 10. It is important to note that we refer to the conformation acquired in this pH range as “native-like”. However, at this stage, and unless we use other biophysical probes that also show the presence of a similar plateau in that range, that name is somewhat arbitrary. The fluorescence spectrum of DGAT3 at pH 8.1 showed a maximum at 345 nm (Figure 1B), indicating that Trp216 and Trp218 dominated the fluorescence of the protein and that both tryptophans were somehow partially buried.

Thermal denaturations at several pHs (3.5, 7.4, and 8.2) were carried out by monitoring the intrinsic fluorescence of DGAT3 after excitation at either 280 or 295 nm (the results were similar at both wavelengths). We did not observe any sigmoidal behavior at any of the explored pHs (Figure S3).

(2) *ANS-binding*—At low pH, the ANS fluorescence intensity at 490 nm was higher and decreased as the pH was raised (Figure S4), suggesting that DGAT3 had solvent-exposed hydrophobic regions at acidic pH values. The intensity at 490 nm showed a sigmoidal-like behavior, but we could not determine its pK_a_-value due to the absence of an acidic baseline. On the other hand, the <λ> of the ANS fluorescence spectra in the presence of DGAT3 (Figure 1A, blank red squares) showed a sigmoidal behavior with a p*K*_a_-value of 6.3 ± 0.6 (fitting the data from pH 4.0 to pH 10.0), which is similar, within error, to that determined by intrinsic fluorescence (Figure 1A, black filled circles). At basic pH values, the <λ> seemed to increase slightly, probably due to the titration of Tyr294, which may have exposed some hydrophobic patches to the solvent.

Thermal denaturations monitored by the emission of ANS at the same explored pH values indicated above did not show any sigmoidal behavior, similar to the thermal denaturation results obtained with intrinsic fluorescence.

In conclusion, the ANS results indicate that the protein has solvent-exposed hydrophobic regions at acidic pH values.

(3) *Examination of tyrosine and tryptophan exposure by fluorescence quenching*—We studied KI quenching in the presence and in the absence of denaturants (Table 1, Figure S5) at different pH values to examine the tertiary structure around Tyr294, Trp216, and Trp218. The tendency either by excitation at 280 or 295 nm was the same. In general, the *K*_sv_ values were larger at acidic and basic pH values, and the *K*_sv_ was smaller at pH 8.1; the largest value, however, corresponded to that when DGAT3 was fully denatured (in 4 M GdmCl, see below). These results indicate that Tyr294, Trp216, and Trp218 are more buried around pH 8., than at acidic or basic pH values, and are fully solvent-exposed under denaturing conditions (Section 3.2).

Taken together, these findings agree with the results from the measurements of the intrinsic and ANS fluorescence, where we had concluded that DGAT3 acquired a “native-like” conformation around pH 8.0, and this conformation was present until pH ~10.

#### 3.1.2. Circular Dichroism (CD)

We only carried out measurements in the far-UV CD, since we had information about the tertiary structure from the fluorescence measurements described above.

**Figure 2:**
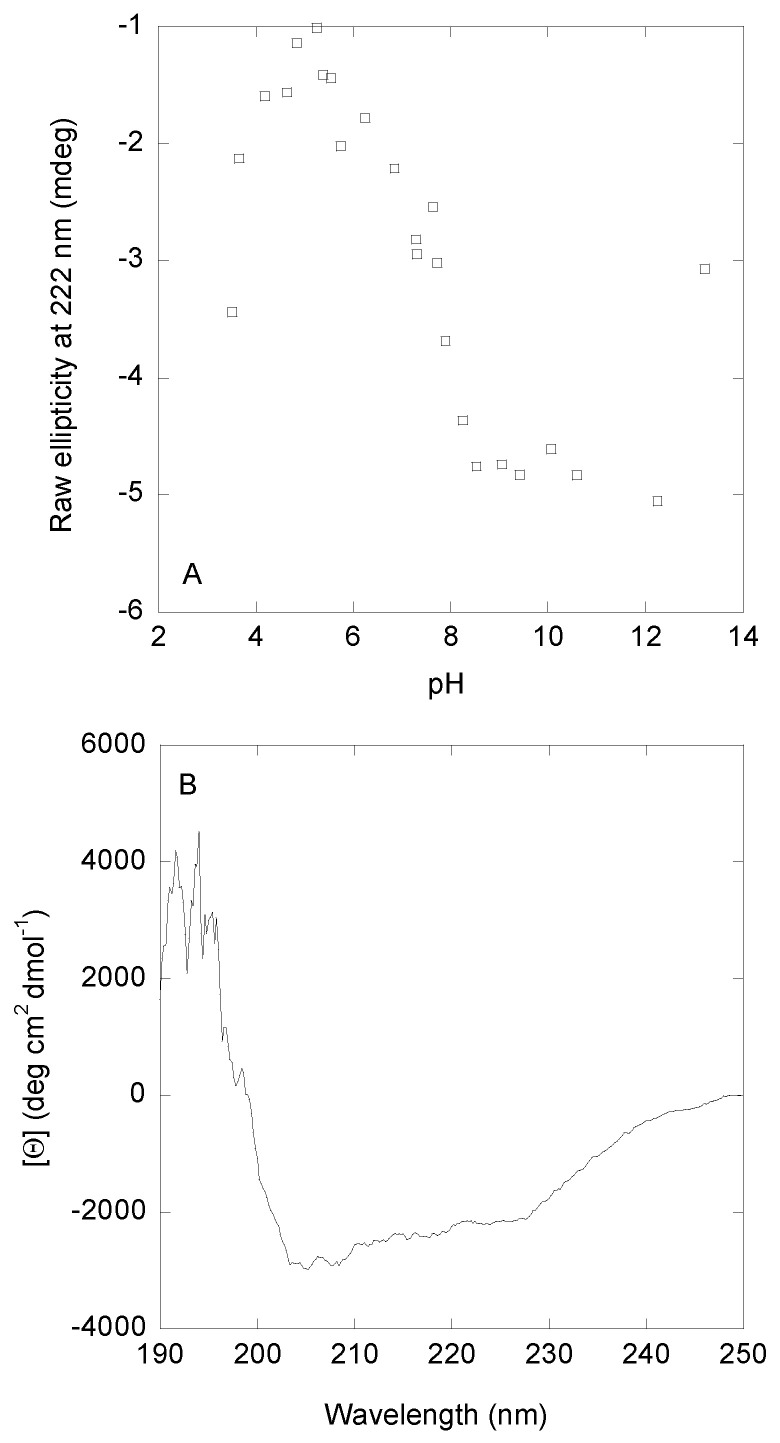
pH-induced conformational changes of DGAT3 followed by far-UV CD. (**A**) Variations in the ellipticity at 222 nm, as the pH of the solution was modified. (**B**) Far-UV CD spectrum of DGAT3 at pH 8.1. Experiments were carried out at 25 °C.

pH titrations monitored by changes in ellipticity at 222 nm showed two main transitions (Figure 2). The first one occurred at acidic pH values until pH ~5.0; however, we could not determine the p*K*_a_-value due to the absence of an acidic baseline. This first transition is the same observed by fluorescence. The second transition was a broad one with a p*K*_a_-value of 6.8 ± 0.6 (obtained by fitting the data in this pH range to Equation (5)), which is similar to those obtained by intrinsic and ANS fluorescence (Figure 1A). Finally, a transition at basic pH values (pH > 12.0) was also observed; this transition, which lacked a basic baseline, started at the same pH values as those monitored by fluorescence (Figure 1A, filled circles), which could correspond to deprotonation of Tyr294. Thus, we can conclude that the acquisition of both secondary and tertiary structure, as monitored by the three probes, occurred concomitantly.

The CD spectrum of DGAT3 at pH 8.0, when the protein acquired a “native-like” conformation, showed two minima at ~210 and ~222 nm (Figure 2B). Since the number of aromatic residues, which also absorb in this region [34,35], in DGAT3 is not very high (1 Tyr, 2 Trp, and 6 Phe), we can safely assume that the shape of the far-UV CD was mainly due to the presence of secondary structure. Deconvolution of such spectrum by using BeStSel [36,37] yielded: 35% of β-sheet, 3% of α-helix; 15% of β-turn, and 47% of disordered conformations.

We also carried out thermal denaturations at the same pH values used in the fluorescence measurements (3.5, 7.4, and 8.2) (Figure S6). As with the fluorescence findings, there was no sigmoidal transition at any of the explored pH values. These findings, together with the results from thermal denaturations followed by fluorescence (intrinsic and ANS), further pinpointed the mainly disordered nature of DGAT3 or, alternatively, a “native-like” conformation that was not stable enough upon heating.

Therefore, we can conclude that: (i) the behavior of the ellipticity at 222 nm was similar to that monitored by intrinsic and ANS fluorescence (Figure 1A), and (ii) at pH 8.0, where the three spectroscopic probes showed acquisition of a “native-like” conformation, deconvolution of the far-UV CD spectrum indicated that its main conformation was that of a disordered polypeptide chain.

### 3.2. The Ordered Structure of DGAT3 Was Unstable

The findings from far-UV CD suggested that DGAT3 at pH ~8.0 contained mainly a high percentage of disordered conformation, but we wondered whether the other ordered conformations (mainly β-sheet from the deconvolution findings) were rigid enough. Our thermal denaturation results described in Section 3.1 indicated that such structures did unfold in a non-cooperative fashion (Figures S3 and S6). Furthermore, we also carried out DSC experiments at pH 8.0, and we did not observe any endotherm in these experiments, but rather a stepwise decrease in the heat capacity upon heating probably due to aggregation (Figure S7). However, we wondered whether the same behavior would be observed for chemical denaturations. Therefore, we carried out urea and GdmCl denaturations using intrinsic fluorescence and far-UV CD at pH 8.0 (50 mM Tris, 150 mM NaCl, and 5 mM TCEP).

#### 3.2.1. Urea Denaturations

**Figure 3:**
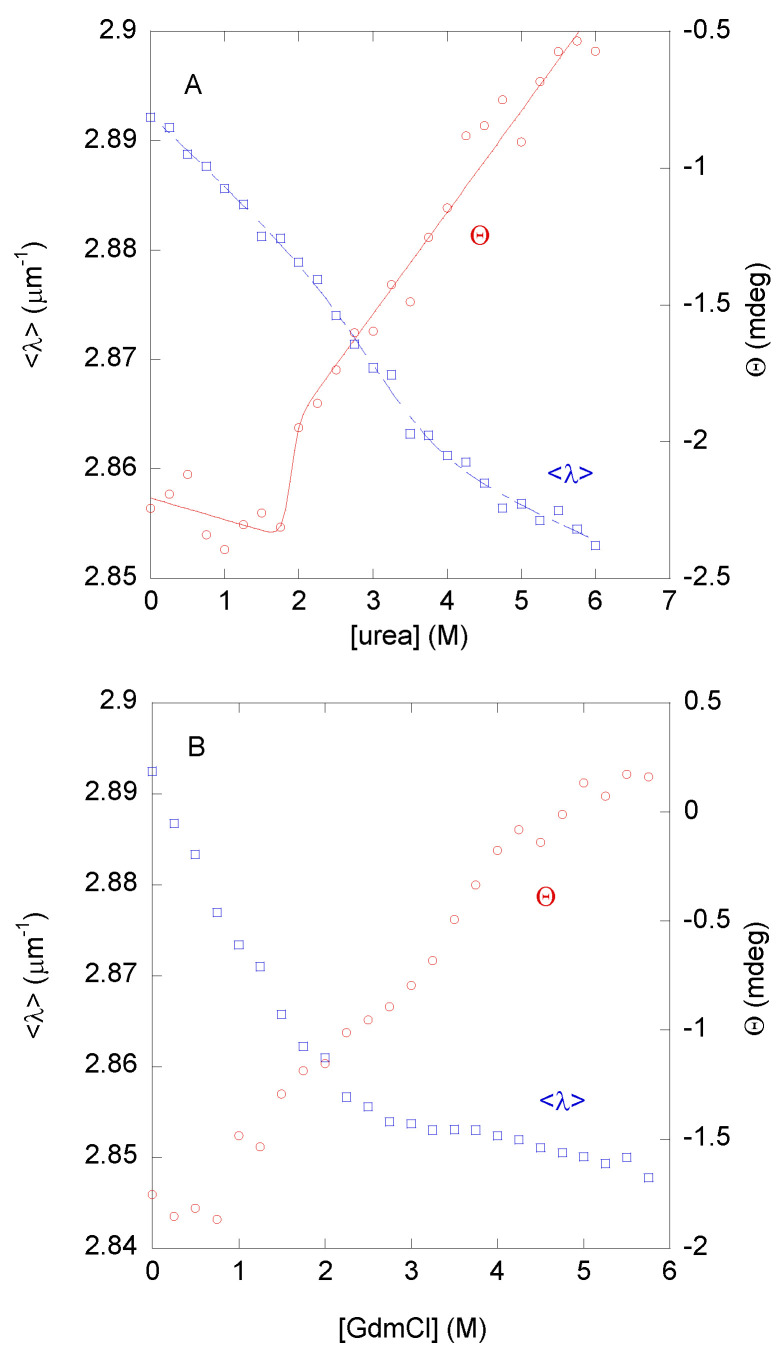
Chemical denaturation of DGAT3. (**A**) The <λ> from the intrinsic fluorescence (blank blue squares, left axis) and the raw ellipticity (blank red circles, right axis) from the spectra of DGAT3 in the urea denaturations. The line through the data represents the fitting to Equation (6) using the LEM. (**B**) The <λ> from the intrinsic fluorescence (blank blue squares, left axis) and the raw ellipticity (blank red circles, right axis) from the spectra of DGAT3 in the GdmCl-denaturations. Experiments were carried out at 25 °C.

The urea denaturations followed by <λ> from intrinsic fluorescence showed a broad transition with a very low *m*-value (1.2 ± 0.5 kcal mol^−1^ M^−1^) and an [urea]_1/2_ = 3.3 ± 0.2 M (Figure 3A, blue blank squares). Conversely, the far-UV CD data also showed a broad transition, having different thermodynamic parameters: *m* = 1.0 ± 0.9 kcal mol^−1^ M^−1^ and [urea]_1/2_ = 1.8 ± 0.1 M (Figure 3A, red blank circles). The urea denaturations were reversible at this pH.

The fact that we observed a sigmoidal transition, although it was very broad, suggested that: (i) the population of well-ordered structure unfolded co-operatively against urea; and (ii) the urea-unfolding of DGAT3 was not two-state, since there was no agreement between the thermodynamic parameters obtained from two different spectroscopic probes, namely CD and fluorescence [38]. These results imply that: (i) there were several partially folded species with different kinds of structure; and (ii) those species were also present in the conformational ensemble of DGAT3 under native conditions due to the Boltzmann equation. From the above values, the Δ*G* of those conformations, which were being unfolded by the presence of urea, was very low: in the range of 2 to 4 kcal mol^−1^ (depending on the values of each spectroscopic technique used for the calculation). These figures, although an estimation of the true protein stability, suggested that the “native-like” conformation of DGAT3 was not very stable.

#### 3.2.2. GdmCl Denaturations

We wondered whether the low cooperativity and the estimates of the low stability observed in the urea denaturations, followed by fluorescence and CD, could be due to the chemical agent used in the denaturation or mirrored the inherent low stability of the folded conformations of DGAT3. To that end, GdmCl was used as the chemical denaturant agent. In this case, the <λ> (at both excitation wavelengths) showed a sigmoidal behavior (Figure 3B, blank blue squares), but no baseline for the folded state (at low GdmCl concentrations) was observed, precluding the fitting of data and the determination of the *m*- and the [GdmCl]_1/2_-values. In addition, the data suggested that the protein was fully unfolded at a [GdmCl] = 4 M, which is why we carried out our quenching experiments at this denaturant concentration (Table 1). On the other hand, the changes in the ellipticity at 222 nm did not show a sigmoidal behavior, but rather a continuous variation of the ellipticity at 222 nm (Figure 3B, blank red circles). Furthermore, these results confirmed that the unfolding reaction of DGAT3 was not two-state, and that the conformational stability of the residual “native-like” conformations of DGAT3 was not very high.

To sum up, we conclude that the small population of well-folded DGAT3 did not have a cooperative unfolding behavior when subjected to heat, but it showed a sigmoidal denaturation curve when treated with chemical agents. However, those partially folded species displayed apparent low stability, as determined by the chemical denaturation findings.

### 3.3. Interaction of DGAT3 with Membrane Models

Our next interest was to evaluate whether DGAT3 interacted with lipids when it acquired a “native-like” conformation at pH 8.0. The possible existence of such interaction would also provide a validation of the protocol used to refold and isolate DGAT3 from inclusion bodies. To test this hypothesis, we used several approaches, namely steady-state anisotropy, fluorescence quenching, and the permeability of liposomes to DGAT3.

#### 3.3.1. Steady-State Fluorescence Anisotropy

**Figure 4:**
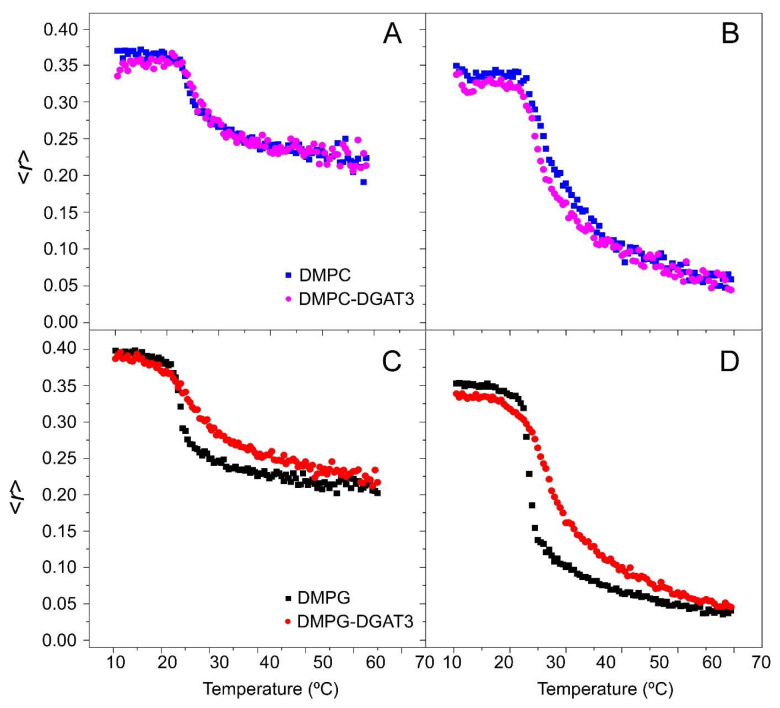
Anisotropy measurements of DGAT3. The steady-state anisotropy <*r*> of TMA-DPH (**A**,**C**) and DPH (**B**,**D**) incorporated into (**A**,**B**) DMPC, and (**C**,**D**) DMPG model membranes as a function of temperature. Data correspond to vesicles containing pure phospholipid and phospholipid plus DGAT3. Experiments were carried out at 25 °C.

The impact of DGAT3 on the structural and thermotropic characteristics of phospholipid membranes was investigated by measuring the steady-state fluorescence anisotropy of DPH and TMA-DPH probes incorporated into zwitterionic DMPC and anionic DMPG membrane models across a range of temperatures (Figure 4). Although the *C. reinhardtii* plasma membrane has no detectable levels of PC, which is thought to be replaced by diacylglyceryl-N,N,N-trimethylhomoserine (DGTS)—a phosphorus-free betaine lipid—both are zwitterionic and bear similar properties [39]. The diphenylhexatrienyl moiety of DPH is situated in the center of the bilayer (inner probe), whereas the TMA-DPH probe extends into the lipid bilayer, specifically between the C-5/C-11 positions of the acyl phospholipid chains (interphase probe), thus providing structural information about these regions and pinpointing the use of both probes to address structural order in lipid bilayers [40,41].

In the case of zwitterionic DMPC membranes, the presence of DGAT3 did not have any effect on the anisotropy of both DPH and TMA-DPH at temperatures both below and above the transition temperature of the phospholipid phase. Furthermore, DGAT3 also failed to influence the cooperativity of the gel-to-liquid crystalline phase transition of the lipid (Figure 4A,B). On the other hand, for anionic DMPG membranes, DGAT3 decreased the cooperativity of the crystalline gel-liquid phase transition of the lipid (Figure 4C,D). Notably, the observed alterations on the DPH and TMA-DPH fluorescence anisotropy curves were analogous, indicating that the interaction of DGAT3 with the lipid membrane models was not located exclusively at a certain depth within the membrane.

**Figure 5:**
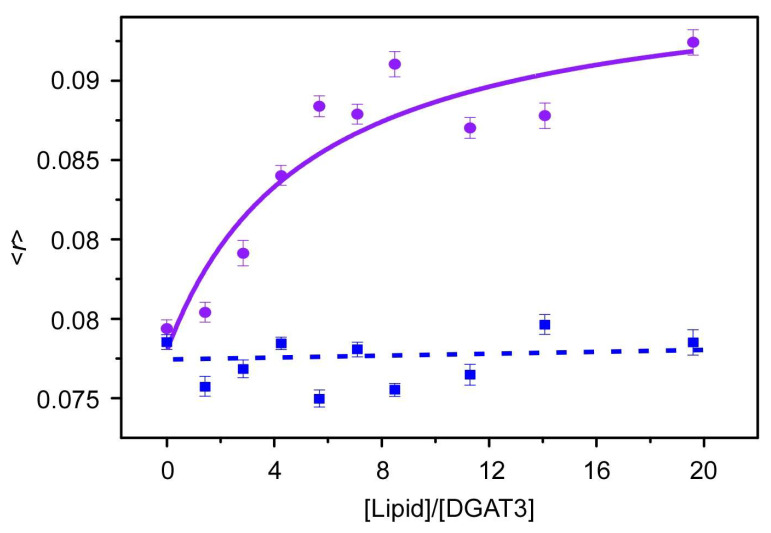
POPC- and POPG-dependent anisotropy measurements. Steady-state anisotropy from DGAT3 as a function of [lipid]/[protein] ratio where the lipid is POPC (blue) and POPG (purple). Excitation and emission wavelengths were 290 and 340 nm, respectively. The solid line for POPG represents the fitting of the data to Equation (3). The dotted line is drawn as a guide to the eye. Experiments were carried out at 25 °C.

Fluorescence anisotropy measurements provide valuable insights into lipid–protein interactions. In solution, the anisotropy value of DGAT3 was in the range 0.075 to 0.08. However, when anionic POPG vesicles were added, this <*r*> value increased as the [lipid] was raised, indicating protein interaction with the membrane. Thus, at high [lipid]/[protein] ratios, the <*r*> approached a limiting value of 0.095 (Figure 5); this higher value indicated a reduction in the mobility of the tryptophans in the presence of POPG membranes [28]. The value of the affinity of this binding was moderate (*K*_p_ ~40,000) [29]. Although the variation in tryptophan anisotropy was relatively modest in the presence of POPG, no significant change in tryptophan anisotropy was observed when zwitterionic POPC model membranes were employed (Figure 5, blue dotted line was drawn to guide the eye). This result shows that there was no DGAT3/lipid interaction when POPC was used.

#### 3.3.2. Fluorescence Quenching

The ability of tryptophans to access the lipid bilayer was evaluated by measuring its fluorescence quenching using KI. If tryptophans were inserted into the hydrophobic core of the bilayer, they would be less susceptible to the effects of the soluble quencher. Therefore, a low level of quenching would indicate that the fluorophore was buried in the hydrophobic core of the bilayer [28].

**Figure 6:**
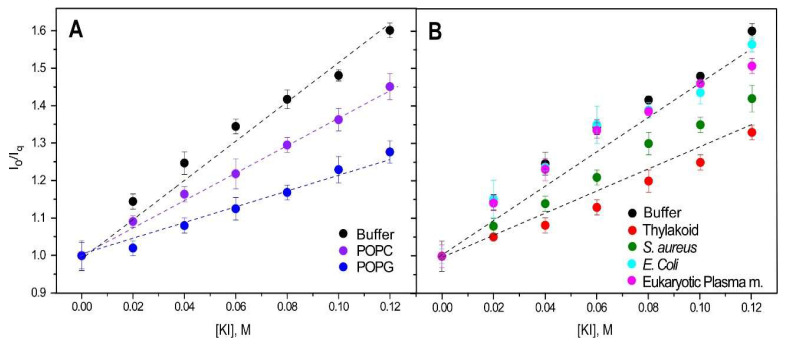
Stern–Volmer plots. Quenching of tyrosine and tryptophans from DGAT3 by KI incorporated into LUVs of POPC or POPG (**A**) or different membrane models (**B**). The dotted lines are drawn to guide the eye. Excitation and emission wavelengths were 275 and 320 nm, respectively. Experiments were carried out at 25 °C.

We used a range of synthetic lipids, including zwitterionic (POPC) and anionic (POPG), as well as model membranes with diverse lipid compositions. These included *E. coli* and eukaryotic plasma membranes, which have a high zwitterionic lipid content, and thylakoid and *S. aureus* membranes, which have a high anionic lipid content. Relative to the control (i.e., isolated DGAT3 in buffer), the quenching by KI was lower when DGAT3 was with POPG or POPC liposomes, although the effect was more noticeable for POPG (Figure 6A). When DGAT3 was incubated with model membranes, there was a significant reduction in quenching in the presence of liposomes (i) mimicking thylakoid membranes (where the light-dependent reactions of photosynthesis occur), and (ii) from *S. aureus* membranes (Figure 6B). On the other hand, there was no significant effect on quenching when the protein was in the presence of liposomes mimicking *E. coli* or eukaryotic plasma membranes (Figure 6B).

To summarize, DGAT3 tryptophans were less accessible to I^-^ in the presence of anionic lipid vesicles, suggesting that Trp216 and Trp218 were protected from the quencher by membrane association.

#### 3.3.3. Impact of DGAT3 on the Permeability of Liposomes

**Figure 7:**
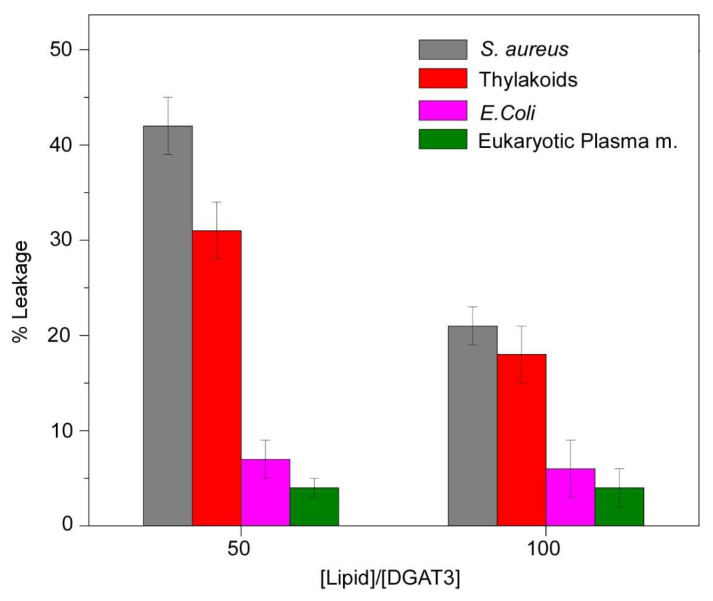
Leakage experiments. The leakage data for LUVs composed of different membrane models were obtained in the presence of DGAT3. The experiment was conducted at 25 °C at two different [protein]/[lipid] ratios.

The permeability of the membrane in the presence of DGAT3 was evaluated through the observation of the leakage of CF, which was entrapped within LUVs. The release of CF from liposomes was observed to be dependent on the lipid composition of the vesicles, with DGAT3 inducing the release with varying effectiveness. Figure 7 shows the outcomes of the experiments conducted with four distinct liposomes, namely, eukaryotic plasma membrane, *E. coli*, *S. aureus,* and chloroplast thylakoid membranes. The leakage was markedly higher for liposomes formed by *S. aureus* and thylakoid lipids than for those composed of eukaryotic plasma and *E. coli* membranes (Figure 7). These results agree with the data obtained with <*r*> and quenching with liposomes, suggesting that DGAT3 indeed interacted with negatively charged lipids.

## 4. Discussion

### 4.1. pH-Denaturation of DGAT3

Although DGAT3 was obtained from inclusion bodies of *E. coli*, the procedure developed based on an on-column refolding worked well, as shown by the fact that the obtained protein was capable of binding to lipids. Thus, the presence of: (i) a low, poorly stable amount of secondary structure; and (ii) a high content of disordered structure, as shown by the biophysical and spectroscopic results, was not a consequence of the refolding protocol, but rather an intrinsic feature of the DGAT3 sequence, which might be important for its function.

DGAT3 acquired its “native-like” conformation at pH ~8.0, which was only present in a narrow pH range. We have called the percentage of secondary structure observed at pH ~8.0 (and obtained by far-UV CD deconvolution) “native-like”, although it cannot be considered that such a structure had rigid hydrogen bonds and was stable for very long times (see below in this section). Rather, we have intended to call “native” or “native-like” the conformation that: (i) was acquired by DGAT3 when the different biophysical probes concomitantly attained a plateau, and (ii) was capable of binding to lipids. In addition, DGAT3 attained its “native-like”, non-rigid, tertiary and secondary conformations in a concomitant manner (Figure 1 and Figure 2). Such acquisition occurred simultaneously with the burial of solvent-exposed hydrophobic patches, as tested by ANS fluorescence. At low pH (pH < 4.0), where there was evidence of secondary structure due to the high (in absolute value) ellipticity at 222 nm, DGAT3 seemed to populate a molten-globule-like species, with non-cooperative thermal transitions (Figures S4 and S5) [33,42]. At this stage, however, we do not know whether that larger percentage of secondary structure (because of the larger helicity) at low pHs was native-like.

The transition observed at low pH, with a p*K*_a_ < 5.0, could be associated with at least one of the several acidic amino acids present in DGAT3 [43]: in the natural primary structure of DGAT3 (Figure S1), there are 18 Asp and 22 Glu, which is 10% of the total number of residues (357). The second transition, which was observed by the three probes resulting in a p*K*_a_ ~6.5, could be attributed to at least one of the nine histidines [43] of the natural sequence of the protein (Figure S1). It could be thought that the His-tag used for purification, and located at the N terminus, could be responsible for the p*K*_a_ observed by following intrinsic fluorescence (Figure 1A); however, we think that this possible behavior is rather unlikely because the tag is located at the N-terminus, very far away from the two tryptophans and the single tyrosine (at the C terminus of the protein) (Figure S1). We cannot rule out that the broad transition observed by far UV CD (Figure 2A), which also shows a p*K*_a_ close to 6.5, could be due in part to the titration of some of the histidines in the purification tag. Finally, the variations at very basic pHs could be attributed to titration of Tyr294 [43].

The far-UV CD spectrum at pH 8.1 (Figure 2B) and its deconvolution [36,37] indicated that DGAT3 had mainly a random-coil conformation with a low percentage of secondary structure, as obtained by deconvolution. This small percentage of secondary structure is what we have called above “native-like”, although it cannot be envisioned as a rigid structure. Rather, the backbone of DGAT3 must explore several short-lived folded conformations within very short time scales to yield such far-UV CD spectrum; in fact, it is interesting to pinpoint that far-UV CD spectra with high percentages of secondary structure (larger than those reported in this work) have been observed in fully unfolded proteins (see, for instance [44]). To further support the deconvolution results of the far-UV CD spectrum, we carried out predictions of disorder based on the primary structure of DGAT3 (Figure S1) by using flDPnn2 [45]; the server predicted five different disordered regions having more than three residues: 1–21, 29–65, 82–85, 148–164, and 329–357. Thus, the number of amino acids in disordered polypeptide patches accounted for 109 residues out of 357, which is 30% of the total number of residues, very close to the percentage of disordered structure from deconvolution of the far-UV CD spectrum (Figure 2B) by using BeStSel [36,37]. These predictions of disorder further pinpoint the fact that the small populations of “native-like” (secondary and tertiary) conformations in DGAT3, as obtained by deconvolution of far-UV CD spectra, are not rigid and are probably short-lived.

The range in which DGAT3 had a “native-like” conformation (pH 8–10) agrees with the pH of the chloroplast stroma during light periods. Light-dependent variations in the pH of the two main sub-compartments within the chloroplast of *Chlamydomonas* have been well documented. In the dark, pH is stably maintained at neutral levels but, upon dark-to-light transitions, it rises to alkaline values in the chloroplast stroma (around 8) and decreases to acidic values in the thylakoid lumen (5.5 to 6 depending on the light intensity) (see [46] and references therein). Conversely, during light-to-dark transitions, it is stabilized again to neutral values. The pH of both the stroma and the lumen are tightly regulated, since it is a parameter of great importance for the correct function of photosynthesis. Acidic levels in the lumen are necessary to activate non-photochemical quenching, and, hence, prevent photo-damage of the photosynthetic machinery during light periods. In the stroma, light-dependent alkalization activates fructose diphosphatase and ribulose-1,5-bisphosphate carboxylase, two enzymes involved in the Calvin cycle [47]. In this scenario, the pH at which DGAT3 attained a “native-like” structure in our conformational studies agrees with our previous results showing that DGAT3 mRNA expression is induced shortly after dark-to-light transitions [11]. Moreover, our findings are compatible with our previous proposal that DGAT3 might be in the stroma and participate in DAG acylation and electron transfer related to photosynthesis [9].

### 4.2. DGAT3 Was Mainly Disordered, with a Poorly Stable, Minor Population of Secondary Structure

The small population of DGAT3 with a secondary structure was not very rigid, as suggested by two pieces of evidence. First, DGAT3 did not show any sigmoidal transition when increasing the temperature, as indicated by fluorescence (Figure S3), far-UV CD (Figure S6), and DSC (Figure S7). Second, when the unfolding of DGAT3 could be monitored by using chemical denaturants, several partially folded species (intermediates) appeared [38]. The conformational stability of those species was very small, below that determined for most proteins (by 5–13 kcal mol^−1^) [48,49,50]. Therefore, the stability of the “native-like” conformations of DGAT3, which probably involve at least some of the two tryptophans (because of the quenching results, Table 1, Figure S5), was very low.

The low stability and the presence of a high amount of disordered structure are probably important for DGAT3 function. Disordered proteins have several key properties that allow them to participate in important cellular processes [51]. Several disordered proteins have been characterized in the chloroplast. A high-throughput analysis carried out on a collection of photosynthetic organisms estimated that disordered proteins account for 39.8% of the total proteins in the *C. reinhardtii* chloroplast [52]. One interesting example refers to CP12, a conditionally disordered protein whose conformation depends on the redox state of its four cysteine residues. During light periods, when reducing conditions prevail in the chloroplast, CP12 is fully disordered. Under the oxidizing conditions of the dark periods, its cysteine residues form two disulfide bridges, which increase the stability of several structural elements [53]. This redox-dependent disorder is important for the main function of CP12, which is related to the dark/light regulation of the Calvin cycle [53].

It could be thought that the mostly unfolded nature of DGAT3 was due to the protocol used during its purification (i.e., on-column refolding from inclusion bodies). However, firmly established protocols, such as those used here, have been employed with other enzymes, fully recovering their activity when rescued from inclusion bodies [17,18,19,54]. It is interesting to note that some of those enzymes, which are completely disordered showing a random-coil far-UV CD spectra, are fully active [55].

In this scenario, the inherent flexibility derived from both the low stability and the disordered structure of DGAT3 could allow it to interact with several macromolecules under different environments within the chloroplast and in different conditions, particularly related to changes in pH or redox potential that dominate after dark-to-light transitions.

### 4.3. DGAT3 Interacted with Anionic Lipid Vesicles

Three different approaches have shown that DGAT3 interacted with lipids. This interaction is proof of the “native-likeness” of DGAT3 under these conditions and provides an explanation for the effectiveness of the on-column refolding procedure used during DGAT3 purification. We can further conclude that DGAT3 had a strong membrane-destabilizing activity, and that the mechanism of action was dependent on the composition of the lipid bilayer. Our results have shown that, when incubated with mono-lipid vesicles, DGAT3 interacted preferentially with anionic POPG compared to zwitterionic POPC liposomes. Furthermore, DGAT3 was also bound to anionic *S. aureus* membranes (100% anionic lipids), whereas no significant association occurred with membranes in which zwitterionic lipids prevailed, such as those of *E. coli* (80% non-anionic lipids) and eukaryotic plasma membrane (92% non-anionic lipids). DGAT3 also interacted with vesicles that mimicked the lipid composition of the thylakoid membranes. Thylakoids do not contain zwitterionic lipids, and have POPG and SQDG as anionic components, although they are not a majority since MGDG and DGDG are uncharged and account for 70% of the total lipids (40% MGDG, 30% DGDG, 16% SQDG, and 14% POPG). In this scenario, future experiments will be carried out to test whether DGAT3 binds preferentially to these two anionic lipids or whether the interaction is a mere consequence of their charge.

The interaction of DGAT3 with PG and the thylakoid lipids is consistent with the chloroplast localization of this protein. In *C. reinhardtii* extra-chloroplast membranes, zwitterionic DGTS and PE are prominent, whereas chloroplast thylakoids contain MGDG, DGDG, SQDG, and PG [56], as stated above. Furthermore, DGAT3 association with anionic lipids and the narrow pH range of its “native-like” conformation were consistent with its likely localization in the stroma of this organelle. Although the major multi-component complexes of the chloroplast thylakoids (photosystem I [PSI], photosystem II [PSII], and cytochrome b_6_f [cytb_6_f]) contain at least small proportions of those four lipids, each one of them has a specific role in thylakoid membrane biogenesis and photosynthesis depending on the nature of its head group [57]. PG and SQDG are, at least in part, functionally redundant, and this might be related to the conservation of an anionic charge on the surface of the thylakoid membrane [58]. Interestingly enough, mutant analyses performed in *C. reinhardtii* and other photosynthetic organisms assign roles for both PG and SQDG in electron transport related to PSII, without a major role in PSI activity [59,60]. In the context of these findings, future experiments will be aimed at studying a possible relationship between DGAT3 and photosystem activity, particularly PSII.

We do not know at this stage whether DGAT3 binding to lipids induced a drastic change in those small populations of folded conformations detected by deconvolution of the far-UV CD spectra by altering the amount of each of those populations. We do not know whether the complex DGAT3/lipid is a fuzzy complex, where the protein remains disordered, as happens in other complexes of intrinsically disordered proteins [55]. Attempts to carry out far-UV CD experiments in the presence of LUVs were hampered by the large scattering of the samples. However, the binding to lipids did not change either the fluorescence emission spectrum or the fluorescence lifetime of the protein, suggesting that the DGAT3 structure did not undergo a severe change, at least in the regions near the tryptophan residues.

## 5. Conclusions

DGAT3 is a novel member of the DGAT family with chloroplast localization and remarkable sequence characteristics. Its study is relevant to advancing our understanding of TAG synthesis in plants and algae. In this work, we have described the conformational features, stability, and interactions with lipids of DGAT3 from *C. reinhardtii*. Our findings demonstrate that DGAT3 exists predominantly in a disordered state, showing only transient, low-stability folding within a narrow alkaline range (pH 8 to 10). Furthermore, we established a clear preference for DGAT3 for anionic lipids, specifically by showing binding to both negatively charged vesicles and membranes that mimic the lipid composition of the thylakoids. We believe that this work provides a basis for advancing knowledge of non-canonical pathways for the synthesis of TAGs in photosynthetic organisms, particularly related to TAG production in the chloroplast in coordination with photosynthetic activity. Moreover, as DGAT3 expression occurs during photosynthetic growth and does not require stress conditions, it is a promising target for future studies oriented to the industrial applications of TAGs. In this scenario, our results will allow the design of a more efficient and stable protein.

## Figures and Tables

**Table 1:** Quenching parameters in KI for DGAT3 ^a^.

Conditions	280	295
pH 3.3	5 ± 2	2.1 ± 0.1
pH 8.1	1.5 ± 0.7	1.82 ± 0.07
pH 12.2	11 ± 2	2.7 ± 0.1
GdmCl ^b^	20 ± 8	2.88 ± 0.08

^a^ Errors are fitting errors to Equation (1). The *K*_sv_ values were obtained by fitting of fluorescence intensity at 315 nm vs. concentration of quenching agent. Experiments were carried out at 25 °C. ^b^ The quenching experiments in the presence of GdmCl were carried out at pH 8.1.

## Data Availability

Data are available from the corresponding authors upon reasonable request.

## Supplementary Materials

The following supporting information can be downloaded at: https://www.mdpi.com/article/10.3390/biom15020245/s1, Figure S1: Seven figures are included: the sequence of the protein; Figure S2: The changes in fluorescence intensity at 330 nm (by excitation at 280 and 295 nm), as the pH was varied; Figure S3: Thermal denaturations followed by fluorescence at different pHs; Figure S4: The changes in the ANS intensity at 480 nm as the pH was varied; Figure S5: Quenching (Stern–Volmer plots) by using KI at different conditions; Figure S6: Thermal denaturations followed by CD at different pHs; Figure S7: DSC experiments carried out at pH 8.0.

## Abbreviations

The following abbreviations are used in this manuscript:

ANS	8-anilinonapthalene-1-sulfonic acid
CD	circular dichroism
CF	carboxifluorescein
CHO	ovine wool cholesterol
CL	18:1 cardiolipin
DAG	1,2-diacylglycerol
DGAT	1,2-diacylglycerol acyltransferase
DGDG	plant digalactosyldiacylglycerol
DGTS	diacylglyceryl-N,N,N-trimethylhomoserine
DMPC	dimyristoylphosphatidylcholine
DPH	1,6-diphenyl-1,3,5-hexatriene
DMPG	dimyristoylphosphatidylglycerol
DSC	differential scanning calorimetry
GdmCl	guanidine hydrochloride
IPTG	isopropyl-β-D-1-thiogalactopyranoside
LB	Luria-Bertani
LEM	linear extrapolation model
LUV	large unilamellar vesicles
MGDG	plant monogalactosyldiacylglycerol
MLV	multilamellar vesicles
PE	phosphatidylethanolamine from bovine liver
PG	L-α-phosphatidylglycerol (from soy) (sodium salt)
PC	L-α-phosphatidylcholine (from egg chicken)
POPC	1-palmitoyl-2-oleoyl-glycero-3-phosphocholine
POPG	1-palmitoyl-2-oleoyl-sn-glycero-3-phospho-(1′-rac-glycerol) (sodium salt)
PS	L-α-phosphatidylserine from porcine brain
SDS	sodium dodecyl sulphate
SM	sphingomyelin (from egg chicken)
SQDG	sulphoquinovosyldiacylglycerol
TAG	triacylglycerol
TCEP	tris(2-carboxyethyl)phosphine
TMA-DPH	1-(4-trimethylammoniumphenyl)-6-phenyl-1,3,5-hexatriene
UV	ultraviolet
