# Supplementary material for: The Diacylglycerol Acyltransferase 3 of Chlamydomonas reinhardtii Is a Disordered Protein Capable of Binding to Lipids Derived from Chloroplasts
Source: Biomolecules. 2025 Feb 8;15(2):245. doi: 10.3390/biom15020245 (PMC11852920; doi:10.3390/biom15020245)
Supplement: Supplementary file 1 [file biomolecules-15-00245-s001.zip › biomolecules-3443391-supplementary.pdf]

Supplementary Materials

**MHHHHHHDDDDKASFGLLGRVPTCGELPKCSARSPESLARSGRARAMVPSGFPSVARAS
HMPTHALVDASRPPAVASPVLCSSLRKERKKLSKVLKSHRKVLERRLSALTTSSVDVDSPL
LTELLSELKTLRTSLEGQRSAALLCAADDSSDSDSDDDGCDTRSAAALAAQRRAAATSAL
TAAASTSQAMTSPARMVVSGLGGQMELTVPELEEGWEWSEADFRAARFEGAPGRVMVCT
GSKCQRKGAQQVLEAVSALADGNTNIEVVPCKCVGKCSAGAALRVRPQGQACATYTQVR
PAQLRDMFEEHFGAVAAAAGAPSAACCVECKSPGSESQPHLHSHAEGAAVEQHVHQLVA**

Figure S1. Primary sequence of DGAT3; the His-tag at the N-terminal region of the chain is indicated in red and the N-terminal methionine in purple.

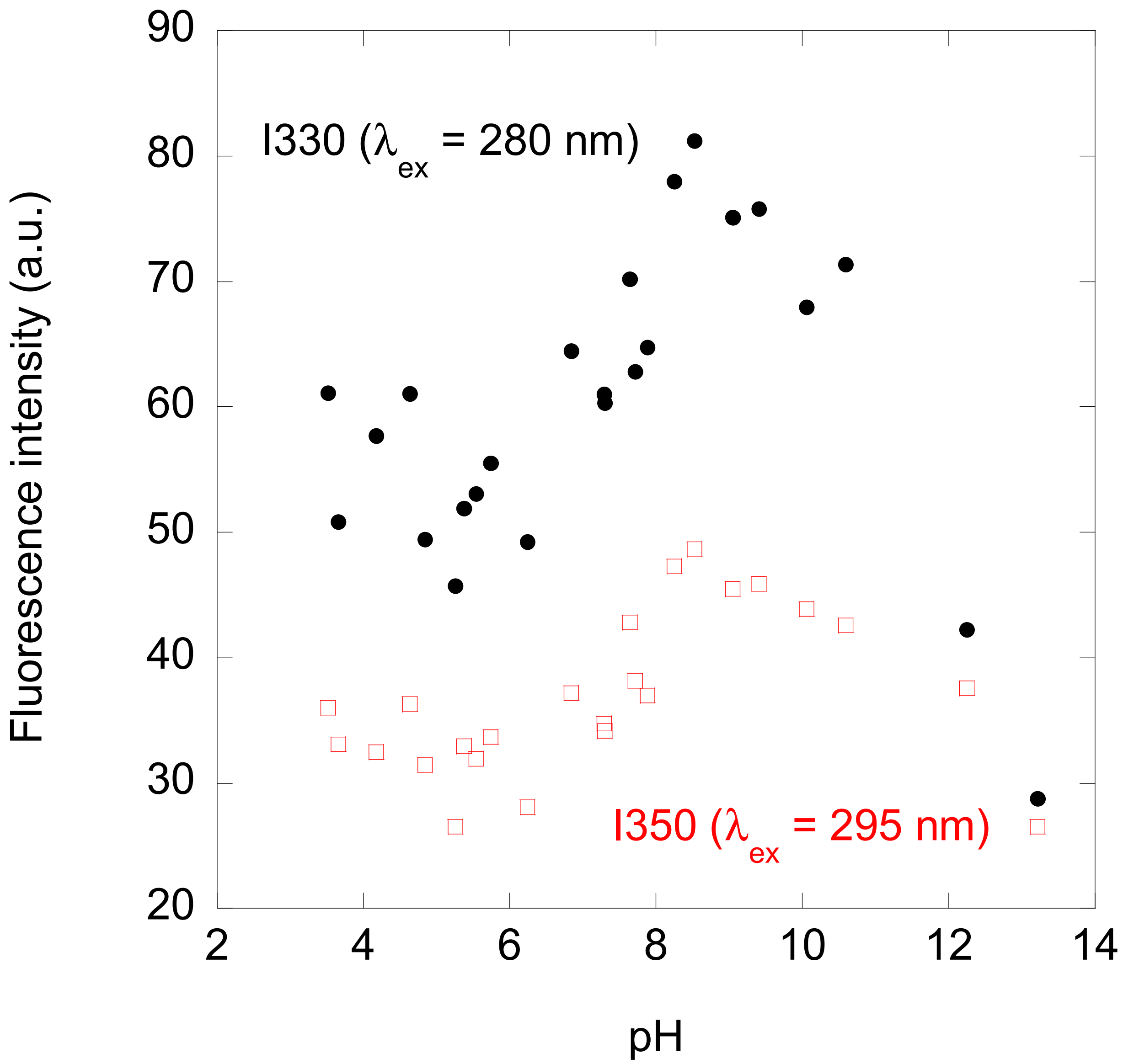

Figure S2. pH-titration of DGAT3 monitored by the changes in two selected wavelengths (330 and 350 nm) at the two excitation wavelengths. Experiments were carried out at 25 ºC.

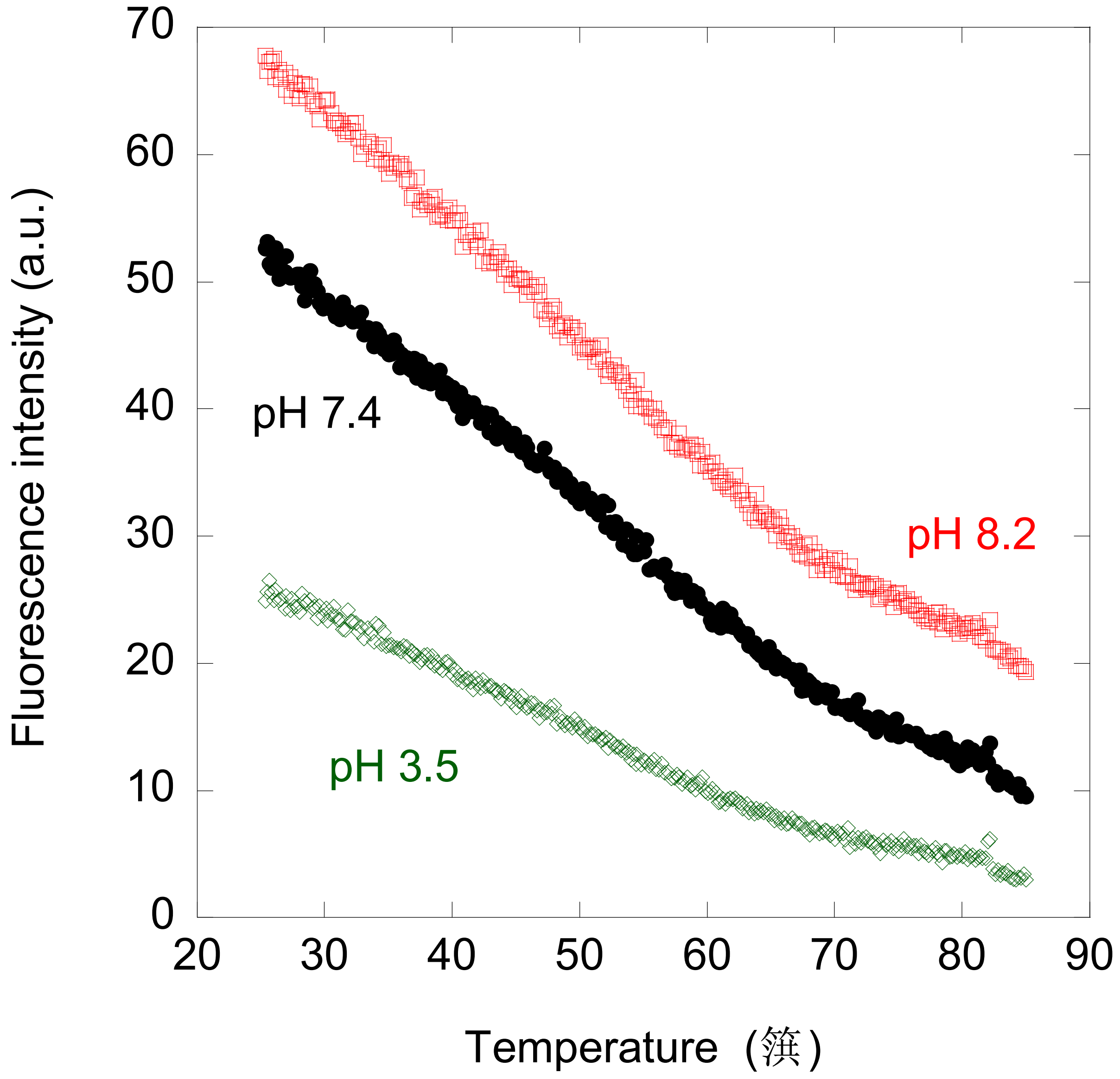

Figure S3. The thermal denaturation of DGAT3 at different pH values after excitation at 280 nm. The wavelength of monitorization of the fluorescence was 330 nm at all pHs. The thermograms have been scaled up for the sake of visual clarity. Similar results were obtained by excitation at 295 nm.

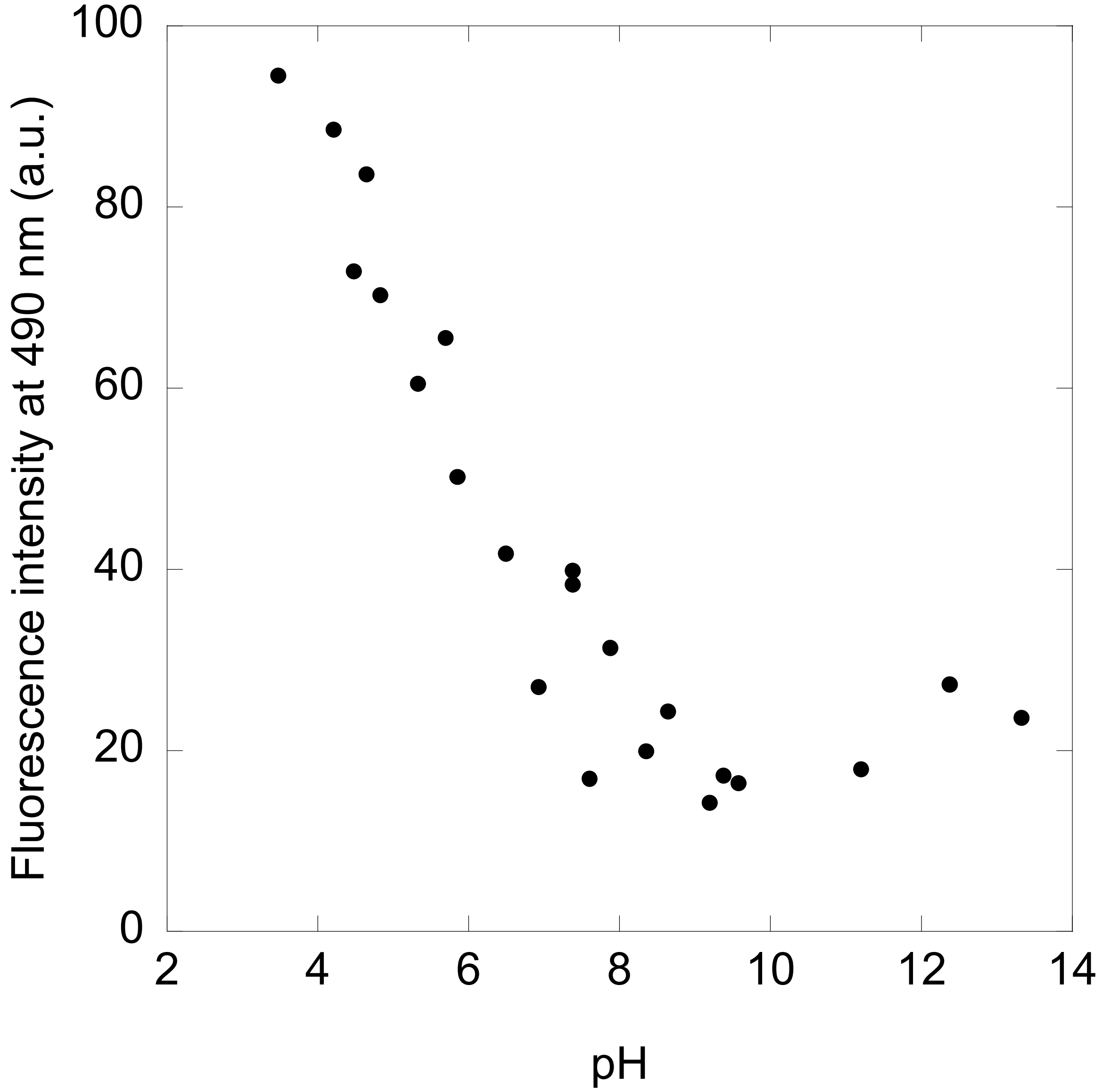

Figure S4. pH-titration of DGAT3 monitored by the changes in the intrinsic fluorescence at 490 nm of the ANS, after excitation at 380 nm. Experiments were carried out at 25 ºC.

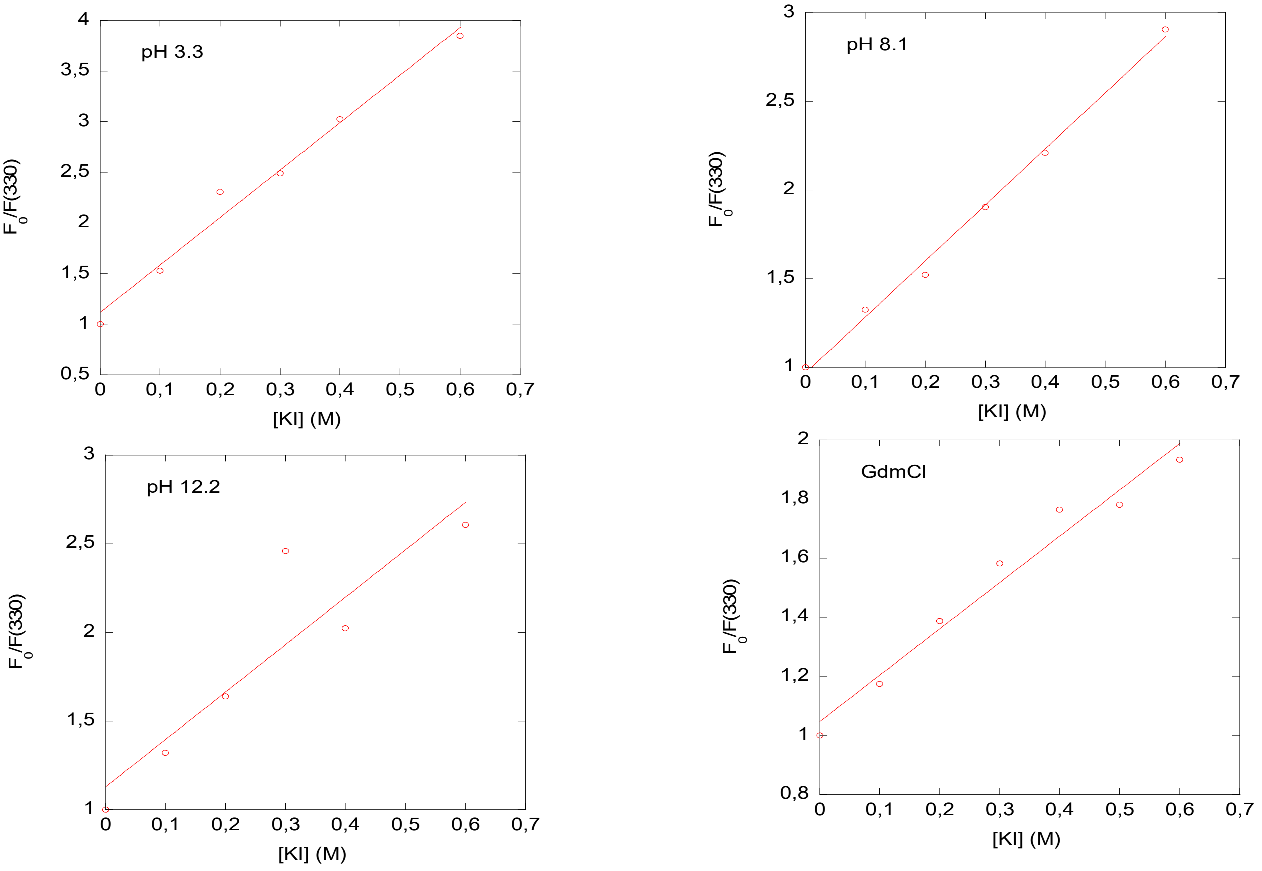

Figure S5. Stern-Volmer plots of the quenching of DGAT3 *in vitro*, at different pH values and conditions.

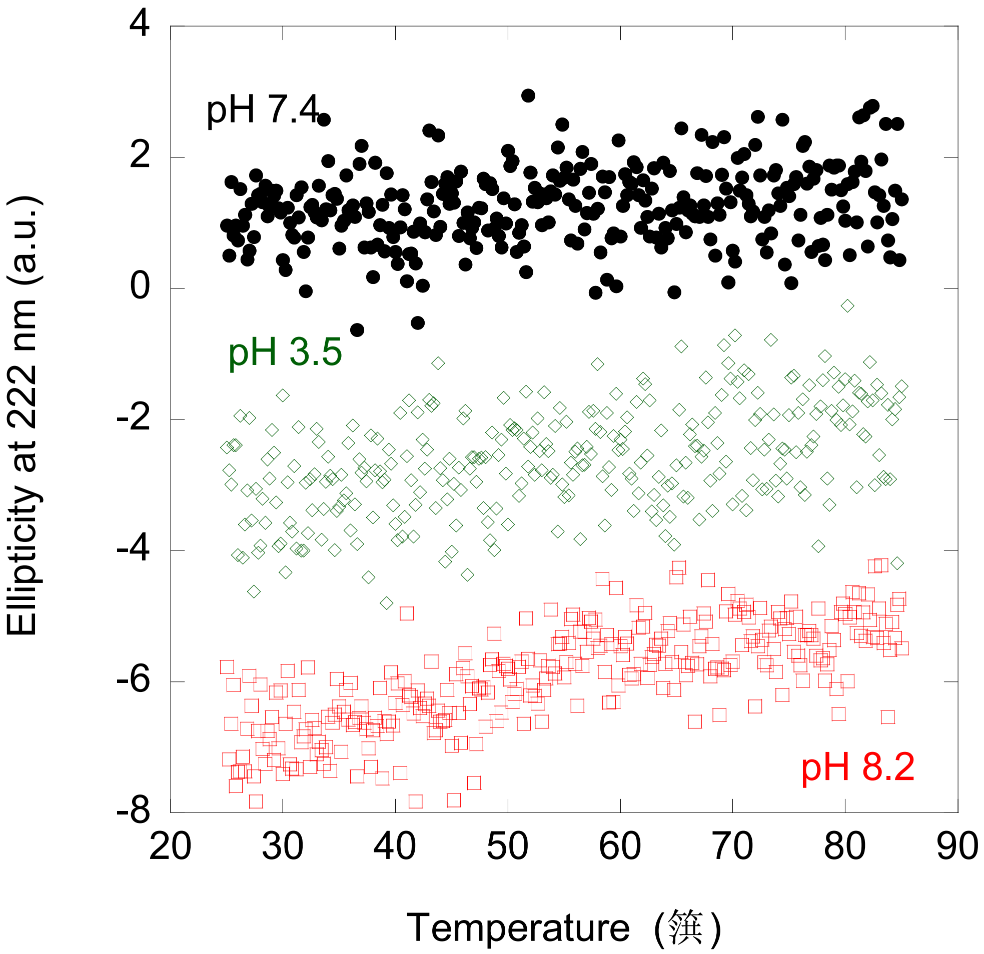

Figure S6. Thermal denaturation of DGAT3 at different pH values followed by the changes in ellipticity at 222 nm (far-UV CD). The thermograms have been scaled up for the sake of visual clarity.

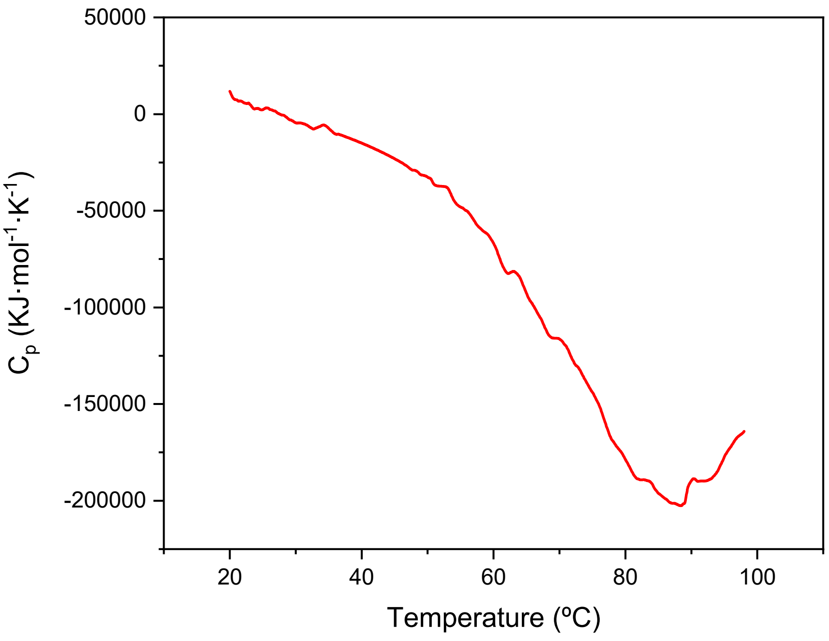

Figure S7. Differential scanning calorimetry of DGAT3: Excess molar heat capacity of DGAT3 as a function of temperature obtained at scan rate of 1.5 °C/min, at pH 8.0.
